# Paroxetine and Mortality in Heart Failure: A Retrospective Cohort Study

**DOI:** 10.3389/fcvm.2021.794584

**Published:** 2022-01-26

**Authors:** Hongxuan Xu, Lingbing Meng, Huanyu Long, Yueping Shi, Yunqing Liu, Li Wang, Deping Liu

**Affiliations:** ^1^Department of Cardiology, Beijing Hospital, National Center of Gerontology, Institute of Geriatric Medicine, Chinese Academy of Medical Sciences, Beijing, China; ^2^The Key Laboratory of Geriatrics, Beijing Institute of Geriatrics, Beijing Hospital National Center of Gerontology, National Health Commission, Institute of Geriatric Medicine, Chinese Academy of Medical Sciences, Beijing, China; ^3^Peking Union Medical College, Chinese Academy of Medical Science, Beijing, China; ^4^Songjiang Hospital, Affiliated to Shanghai Jiaotong University School of Medicine (Preparatory Stage), Shanghai, China; ^5^Departments of Neurology, Beijing Hospital, National Center of Gerontology, Institute of Geriatric Medicine, Chinese Academy of Medical Sciences, Beijing, China; ^6^Peking University Health Science Centre, Peking University Fifth School of Clinical Medicine, Beijing, China

**Keywords:** paroxetine, mortality, heart failure, cohort study, retrospective study

## Abstract

**Introduction:**

Paroxetine is a GRK2 inhibitor that has been widely used to treat depression and anxiety over the last few decades. The inhibition of GRK2 has been studied extensively *in vivo*; however, evidence of its impact on heart failure remains scarce.

**Methods:**

To assess the association between paroxetine use and mortality in patients with heart failure. We conducted a retrospective longitudinal cohort study from 2008 to 2019, with a follow-up time of 28 days for all groups. This is a single-center study using the Medical Information Mart for Intensive Care IV database with 11,657 heart failure patients identified. We performed genetic matching to adjust for the covariates. Heart failure patients prescribed paroxetine for >24 h after hospital admission were categorized into the paroxetine group (77 patients), with remaining heart failure patients making up the matched control group (231 patients). The primary outcome was 28-day all-cause mortality from the date of hospital admission. Secondary outcomes included length of intensive care unit stay, length of hospital stay, and in-hospital mortality. The Kaplan–Meier survival estimator, logistic regression, Cox regression, and restricted mean survival time were used to detect the association between paroxetine therapy and outcomes.

**Results:**

Patients who received paroxetine during one hospital admission lived, on average, 0.7 lesser days (95% CI −2.53 to 1.1, *p* = 0.46) than patients who did not use it in a 28-day truncation time point. Multivariable logistic regression, including all matched covariates, demonstrated that the adjusted odds ratio of 28-day mortality of the paroxetine administration group was 1.1 (95% CI 0.37–2.9, *p* = 0.90). Multivariable Cox regression of 28-day mortality presented an adjusted hazard ratio of 1.00 (95% CI 0.42–2.62, *p* = 0.92). Paroxetine was associated with an increased survival time at a 3,000-day truncation time point (203 days, 95% CI −305.69 to 817.8, *p* = 0.37).

**Conclusions:**

In patients with heart failure, treatment with paroxetine did not significantly reduce 28-day all-cause mortality.

## Introduction

Heart failure (HF) often results from different CVDs and other conditions that lead to myocardial and vascular damage. β-adrenergic receptors (β-ARs), which belong to the superfamily of G protein-coupled receptors (GPCRs), regulate cardiac contractility and heart rate in response to catecholamine release. HF patients have higher preload and afterload, resulting in increased cardiac work, which escalates catecholamine release from the adrenal medulla via sympathetic nerve fibers (1). Chronic exposure to catecholamines stimulates GPCR kinase 2 (GRK2) upregulation in the heart. GRK2 protects the heart from catecholaminergic overstimulation in a newly occurring injury (2). However, dysregulated GRK2 expression during established HF leads to agonist-occupied β-AR desensitization and downregulation, diminishing cardiac reserves (3, 4).

Lowering cardiac myocyte GRK2 activity *in vivo* and targeted deletion of GRK2 preserved inotropy, which benefitted the phenotype of established HF. Cardiomyopathic mice expressing βARKct (a cardiac GRK2 inhibitor) exhibited a significant increase in mean survival age, showed less cardiac dilation, improved cardiac function, and left ventricular end-diastolic dimension compared to the control group (2, 3).

### Hypothesis

Paroxetine is a selective serotonin reuptake inhibitor (SSRI) that has been widely used to treat depression and anxiety over the last few decades. It binds to and reorganizes the active site of GRK2, which overlaps with the adenosine and ribose sub-binding sites of ATP (5, 6). In addition, paroxetine has a lower affinity for other GRK receptors. Paroxetine inhibits GRK2-dependent phosphorylation of activated GPCRs, β-arrestin 2 recruitment, and receptor internalization (6). Post-myocardial infarction, mice treated with paroxetine exhibited considerably improved left ventricular function and structure compared to mice treated with fluoxetine (7). This indicates that the cardiac benefits of paroxetine were not due to the effect of its SSRI activity.

The inhibition of GRK2 has been studied extensively *in vivo*, but evidence of its cardiac benefits in humans remains scarce. In a small, randomized study, after 8 weeks of treatment with paroxetine, patients with acute myocardial infarction and depression had a significantly improved LV ejection fraction and reduced circulating catecholamine levels than patients receiving fluoxetine (8).

Based on the previous literature, we postulate that prescribing paroxetine to patients with HF could have mortality benefits.

## Methods

### Study Cohort

We conducted a longitudinal, single-center, retrospective study of HF patients from the medical and surgical intensive care units (ICUs) based on the Medical Information Mart for Intensive Care IV (MIMIC-IV version 1.0) database, MIMIC is a large, freely-available database comprising de-identified health-related data from patients who were admitted to the critical care units of the Beth Israel Deaconess Medical Center (9). We followed the Strengthening the Reporting of Observational Studies in Epidemiology (STROBE) guidelines while reporting this study (10). This MIMIC project was approved by the institutional review boards of the Massachusetts Institute of Technology and Beth Israel Deaconess Medical Center and was granted a waiver of informed consent because the project did not impact clinical care and all protected health information was de-identified.

This study was designed to investigate whether paroxetine administration independently contributes to improving the mortality of patients with HF. We used the ICD-9 and ICD-10 codes to identify specific diseases, and the codes were documented by hospital staff on patient discharge. We used only the data from each patient's first ICU admission in this study. The patients who were prescribed paroxetine for longer than 24 h in the initial prescription after their hospital admission were categorized into the paroxetine group, with the remaining HF patients making up the control group. The code for data extraction is available on GitHub (https://github.com/MIT-LCP/mimic-code).

### Genetic Matching

Genetic matching is a generalization of the propensity score and Mahalanobis distance, developed by Mebane and Sekhon, using an evolutionary search algorithm to maximize the balance of observed covariates across matched treated and control units (11). We performed matching using a genetic matching algorithm as implemented in the Matching package in R (4.1.0) (12). We used the average treatment effect for the treated (ATT) as our matching estimator, because not all eligible HF patients are likely to have undergone paroxetine administration (13). The Matching package implements the matching estimators and standard error estimators described by Abadie and Imbens (14). To improve robustness, we performed bias correction on all continuous covariates using the BiasAdjust option (15).

### Covariates

We extracted patients' demographic data, including age, sex, ethnicity, body mass index, and severity at admission measured by the first 24-h Simplified Acute Physiology Score II.

### Comorbidities

Data on anxiety, depression, acute stress, myocardial infarction, peripheral vascular disease, cerebrovascular disease, dementia, chronic pulmonary disease, rheumatic disease, peptic ulcer disease, mild liver disease, diabetes without complications, diabetes with complications, paraplegia, renal disease, malignant tumor, severe liver disease, metastatic solid tumor, AIDS, and atrial fibrillation were extracted. All comorbidities were identified using the recorded ICD-9 and ICD-10 codes.

### Vital Signs

The mean systolic blood pressure, diastolic blood pressure, mean arterial pressure, heart rate, respiratory rate, and mean SpO_2_ were recorded on the first day.

### Treatments

Treatments included angiotensin-converting enzyme inhibitors, angiotensin receptor blockers, angiotensin receptor-Neprilysin inhibitors, Beta-blockers, and diuretics, digoxin, sodium-glucose cotransporter 2 inhibitors, and SSRIs other than paroxetine.

### Cardiac Markers

Ejection fraction and brain natriuretic peptide levels were used as cardiac markers.

### Primary and Secondary Outcomes

When taken orally, paroxetine achieves maximum concentration in about 6–10 h and reaches steady-state in 7–14 days. Therefore, the primary outcome was 28-day all-cause mortality from the date of hospital admission. Notably, the date of death was extracted only from the hospital information system. Data on out-of-hospital mortality is currently unavailable in MIMIC-IV v1.0. Therefore, we assumed the date of death information up to 150 days after hospital admission. Secondary outcomes included length of ICU stay (LOS ICU), length of hospital stay (LOS hospital), and in-hospital mortality.

### Statistical Analysis

Baseline characteristics are presented as mean (standard deviation) or median (interquartile range) for continuous variables and number (percentage) for categorical variables. We used the *t*-test or Wilcoxon rank-sum test to compare the differences among continuous variables with or without normal distribution. In addition, Pearson's Chi-square (χ^2^) tests were used to compare the differences in categorical variables between the two groups.

We performed 1:3 genetic matching to balance the baseline characteristics between the paroxetine and control groups. After matching, standardized mean differences (SMDs) were used to evaluate the balance of characteristics between the two groups. A variable can be considered a balance when the SMD is <0.1 (16).

Kaplan–Meier (KM) estimation, logistic regression, and Cox regression were performed to detect the association between paroxetine therapy and outcomes. Restricted mean survival time (RMST) analysis was performed to describe the area under the KM survival curve during a pre-specified timepoint.

We performed different matching strategies to test the robustness of our study: propensity score matching, coarsened exact matching, and optimal full matching.

In addition, multivariate imputation was used to impute the missing values under the assumption of missing at random (17). All statistical analyses were performed using R (version 4.1.0). Statistical significance level was defined as *p* < 0.05.

## Results

**Figure 1:**
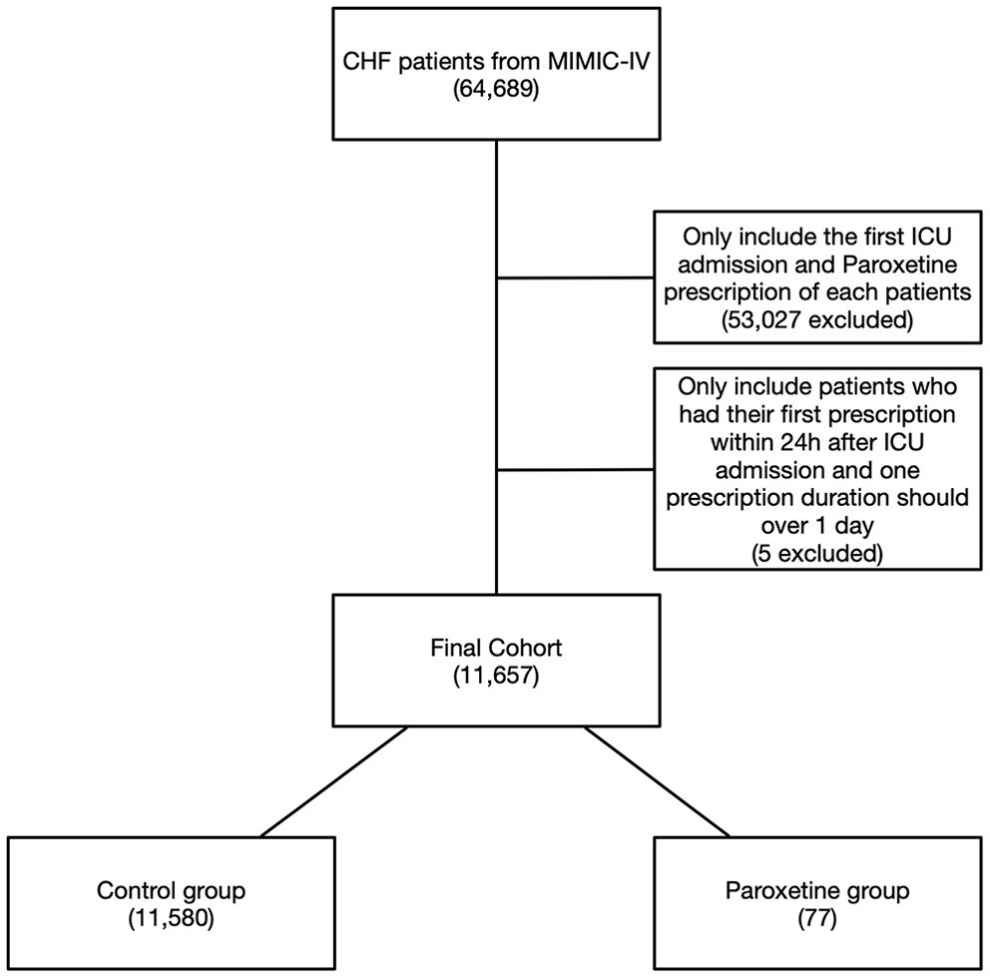
Flowchart of study cohort. Illustration of exclusion and inclusion criteria used to select the final cohort of 11,657 patients. CHF, congestive heart failure; MIMIC-IV, Medical Information Mart for Intensive Care IV.

We identified HF in 64,689 patients after reviewing 382,278 MIMIC-IV admissions, and 11,657 patients were included in the final cohort after identifying the first ICU admission and restricting to paroxetine prescription (Figure 1). Paroxetine was prescribed to 77 (0.7%) patients with HF. The mean paroxetine administration time of one prescription was 5.76 ± 3.51 days. The mean dosage of paroxetine per 24 h was 14.28 mg. The characteristics of the original and post-matching cohorts are summarized in Table 1. The paroxetine group had significantly more females, 51 (66.2%) vs. 5,261 (45.4%), before matching, and no patient had been diagnosed with anxiety, depression, or acute stress. We conducted genetic matching based on the covariates of the demographic data, comorbidities, prescriptions, vital signs, and cardiac markers. After genetic matching, the SMD of all covariates was <0.1, indicating a similar distribution.

**Table 1:** Comparison of the original cohort and the matched cohort.

	**Original cohort**			**Matched cohort**			
	**Control**	**Paroxetine**	***p*** **-value**	**Control**	**Paroxetine**	***p*** **-value**	**Missing data (%)**
*n*	11,580	77		231	77		
**Demographics**							
Age [median (IQR)], year	75.00 [65.00,84.00]	77.00 [65.00,85.00]	0.62	75.00 [67.00,84.00]	77.00 [65.00,85.00]	0.90	0
Ethnicity (non-white) (%)	3,462 ± 29.9	16 ± 20.8	0.11	48 ± 20.8	16 ± 20.8	1.00	0
Gender (male) (%)	6,319 ± 54.6	26 ± 33.8	<0.001	82 ± 35.5	26 ± 33.8	0.89	0
Body mass index [median (IQR)]	27.82 [24.13,32.78]	29.99 [25.97,33.87]	0.05	29.17 [25.00,33.08]	29.99 [25.97,33.87]	0.59	44.21
**Vital signs**							
Heart rate [mean (SD)]	83.96 ± 16.13	83.17 ± 12.64	0.67	82.62 ± 13.32	83.17 ± 12.64	0.75	0.17
Systolic blood pressure [median (IQR)], mmHg	114.08	116.41	0.71	114.38	116.41	0.93	0.47
	[105.12,126.17]	[102.03,129.04]		[105.42,126.78]	[102.03,129.04]		
Diastolic blood pressure [median (IQR)], mmHg	59.79 [53.30,67.46]	58.79 [51.75,65.64]	0.50	59.65 [53.22,66.87]	58.79 [51.75,65.64]	0.78	0.47
Mean blood pressure [median (IQR)], mmHg	74.95 [69.08,82.04]	73.42 [67.48,83.11]	0.307	74.36 [68.02,81.02]	73.42 [67.48,83.11]	0.88	0.17
Respiratory rate [median (IQR)]	19.35 [17.18,22.11]	19.00 [17.59,20.73]	0.637	19.21 [17.78,20.78]	19.00 [17.59,20.73]	0.82	0.19
SpO_2_ [median (IQR)], %	96.75 [95.33,98.09]	96.58 [95.14,97.83]	0.196	96.70 [95.34,97.83]	96.58 [95.14,97.83]	0.60	0.28
SAPSII score [mean (SD)]	39.72 ± 13.41	39.65 ± 14.30	0.964	38.92 ± 11.18	39.65 ± 14.30	0.65	0
**Comorbidities**							
Myocardial infarction (%)	3,689 (31.9)	22 (28.6)	0.621	72 (31.2)	22 (28.6)	0.78	0
Peripheral vascular disease (%)	1,878 (16.2)	9 (11.7)	0.357	21 (9.1)	9 (11.7)	0.66	0
Cerebrovascular disease (%)	1,587 (13.7)	6 (7.8)	0.181	18 (7.8)	6 (7.8)	1.00	0
Dementia (%)	577 (5.0)	2 (2.6)	0.486	6 (2.6)	2 (2.6)	1.00	0
Chronic pulmonary disease (%)	4,303 (37.2)	36 (46.8)	0.106	109 (47.2)	36 (46.8)	1.00	0
Rheumatic disease (%)	525 (4.5)	4 (5.2)	0.997	12 (5.2)	4 (5.2)	1.00	0
Peptic ulcer disease (%)	259 (2.2)	1 (1.3)	0.866	0 (0.0)	1 (1.3)	0.56	0
Severe liver disease (%)	285 (2.5)	2 (2.6)	1.00	3 (1.3)	2 (2.6)	0.80	0
Mild liver disease (%)	949 (8.2)	3 (3.9)	0.244	9 (3.9)	3 (3.9)	1.00	0
Diabetes without complication (%)	3,383 (29.2)	28 (36.4)	0.212	78 (33.8)	28 (36.4)	0.78	0
Diabetes with complication (%)	1,863 (16.1)	12 (15.6)	1.00	35 (15.2)	12 (15.6)	1.00	0
Paraplegia (%)	405 (3.5)	1 (1.3)	0.461	3 (1.3)	1 (1.3)	1.00	0
Renal disease (%)	4,471 (38.6)	35 (45.5)	0.27	90 (39.0)	35 (45.5)	0.38	0
Malignant cancer (%)	1,191 (10.3)	4 (5.2)	0.20	10 (4.3)	4 (5.2)	1.00	0
Metastatic solid tumor (%)	419 (3.6)	0 (0.0)	0.16	0 (0.0)	0 (0.0)	1.00	0
AIDS (%)	50 (0.4)	0 (0.0)	1.00	0 (0.0)	0 (0.0)	1.00	0
Atrial fibrillation (%)	5,666 (48.9)	39 (50.6)	0.85	116 (50.2)	39 (50.6)	1.00	0
Anxiety (%)	78 (0.7)	0 (0.0)	0.98	1 (0.4)	0 (0.0)	1.00	0
Depression (%)	3 (0.0)	0 (0.0)	1.00	0 (0.0)	0 (0.0)	1.00	0
Acute stress (%)	3 (0.0)	0 (0.0)	1.00	0 (0.0)	0 (0.0)	1.00	0
**Treatments**							
ACEI (%)	3,832 (33.1)	34 (44.2)	0.05	96 (41.6)	34 (44.2)	0.79	0
ARB (%)	1,323 (11.4)	11 (14.3)	0.54	29 (12.6)	11 (14.3)	0.85	0
Beta blocker (%)	7,366 (63.6)	53 (68.4)	0.53	155 (67.0)	53 (68.4)	0.95	0
ARNi (%)	54 (0.5)	0 (0.0)	1.00	1 (0.4)	0 (0.0)	1.00	0
Mineralocorticoid receptor antagonists (%)	1,454 (12.6)	8 (10.7)	0.64	25 (10.8)	8 (10.7)	1.00	0
Diuretics (%)	9,480 (81.9)	60 (77.9)	0.46	186 (80.5)	60 (77.9)	0.74	0
Digoxin (%)	1,199 (10.4)	9 (11.7)	0.85	27 (11.7)	9 (11.7)	1.00	0
SGLT2i (%)	2 (0.0)	0 (0.0)	1.00	0 (0.0)	0 (0.0)	1.00	0
SSRi other than Paroxetine (%)	5 (0.04)	0 (0.0)	1.00	0 (0.0)	0 (0.0)	1.00	0
**Cardiac function**							
Brain natriuretic peptide (tested) (%)	1,042 (9.0)	3 (3.9)	0.17	9 (3.9)	3 (3.9)	1.00	0
Ejection fraction (tested) (%)	0 (0)	0 (0)	1.00	0 (0)	0 (0)	1.00	0

*ACEI, angiotensin-converting enzyme inhibitors; ARBs, angiotensin receptor blockers; ARNi, angiotensin receptor-neprilysin inhibitors; IQR, interquartile range; SAPSII, Simplified Acute Physiology Score II; SD, standard deviation; SGLT2i, sodium-glucose cotransporter 2 inhibitors; SSRI, selective serotonin reuptake inhibitor*.

### 28-Day Mortality

**Figure 2:**
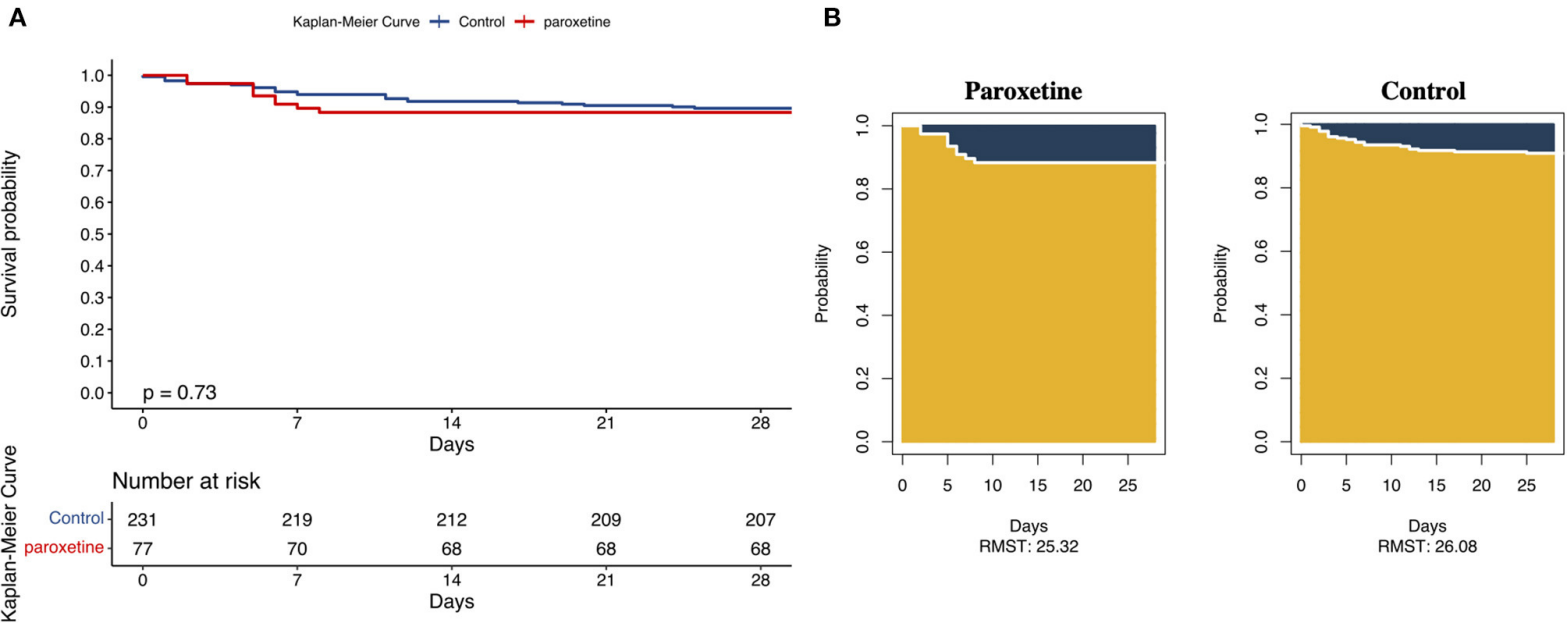
**(A)** Kaplan–Meier Curves and Restricted Mean Survival Time (RMST) for the Incidence of 28-Day All-Cause Death. This was a 1:3 matched cohort. The follow-up time was 28 days in both the groups. **(B)** The RMST for the primary endpoint (all-cause death) was 25 days (95% CI, 24–27 days) in the paroxetine group and 26 days (95% CI, 25–27 days) in the control group. There were 24 patients in the paroxetine group with an event and nine in the control group.

The KM curve of 28-day mortality after matching is presented in Figure 2. The difference in RMST can be explained by the difference in areas of the KM curve for the two groups, which indicates the difference in survival time due to the intervention during a pre-specified period. The matched data showed that patients in the paroxetine group were expected to live 25 days (95% CI 24–27) vs. 26 days (95% CI 25–27) in the control group using a 28-day truncation time point (Figure 2). Patients who received paroxetine during one hospital admission lived, on average, 0.7 lesser days (95% CI −2.53 to 1.1, *p* = 0.46) than the control patients over 28 days.

Multivariable logistic regression, including all the covariates matched, demonstrated that the adjusted odds ratio of 28-day mortality of the paroxetine administration group was 1.1 (95% CI 0.37–2.9, *p* = 0.90; Table 2). Multivariable Cox regression of 28-day mortality presented an adjusted hazard ratio of 1.00 (95% CI 0.42–2.62, *p* = 0.92).

**Table 2:** Primary outcome (28-day mortality) analysis with four different match methods.

**Method**	**Unadjusted (95% CI)**	***p*** **-value**	**Adjusted (95% CI)**	***p*** **-value**
**Logistic regression**	**Odds ratio**			
Genetic matching	1.14 (0.48–2.50)	0.75	1.1 (0.42–2.90)	0.79
Propensity score matching	0.89 (0.33–2.30)	0.81	0.93 (0.28–3.10)	0.91
Coarsened exact matching	1.23 (0.32–4.09)	0.74	1.51 (0.32–6.30)	0.55
Optimal full matching	1.23 (0.32–4.09)	0.74	1.51 (0.32–6.30)	0.55
**Cox regression**	**Hazard ratio**			
Genetic matching	1,10 (0.53–2.5)	0.70	1.00 (0.42–2.62)	0.92
Propensity score matching	0.89 (0.36–2.20)	0.80	0.40 (0.04–3.70)	0.42
Coarsened EXACT MATCHING	1.30 (0.39–4.10)	0.70	5.1 (0.52–51.00)	0.16
Optimal full matching	1.30 (0.39–4.10)	0.70	5.1 (0.52–51.00)	0.16

*95% CI, 95% confidence interval*.

As summarized in Table 2, all four methods failed to reveal significantly different results.

### Secondary Outcomes

We have performed our analyses using different end-points, but the results of the Cox regression are similar or identical, such as 56-day mortality and 90-day mortality (HR 1.12, 95% CI 0.35–3.60, *p* = 0.838).

**Figure 3:**
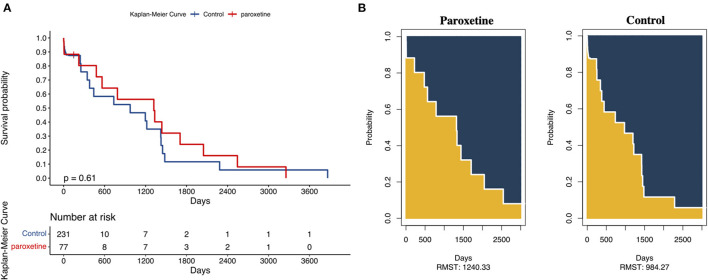
**(A)** Kaplan–Meier Curves and Restricted Mean Survival Time (RMST) for the incidence of 3,000-day all-cause death. | represents a sensor mark 150 days after ICU admission. (B) The RMST for all-cause death was 1,240 days (95% CI, 792–1,688 days) in the paroxetine group and 984 days (95% CI, 645–1,323 days) in the control group. There were 230 patients in the paroxetine group with an event or censorship and 77 in the control group.

We adopted a 3,000-day truncation time point to represent recorded in-hospital mortality. The KM curve of the recorded in-hospital mortality after matching is presented in Figure 3. The paroxetine group was expected to live 1,240 days (95% CI 792–1,688), while the control group was expected to live 984 days (95% CI 645–1,323; Figure 3). Paroxetine was associated with an increased survival time at a 3,000-day truncation time point (203 days, 95% CI −305.69 to 817.80, *p* = 0.37). The adjusted odds ratio of recorded in-hospital mortality was 1.7 (95% CI 0.81–3.30, *p* = 0.16). In addition, multivariable Cox regression analysis of in-hospital mortality presented an adjusted hazard ratio of 0.78 (95% CI 0.40–1.54, *p* = 0.48) (Table 2).

The paroxetine group showed an not significant difference in LOS in the ICU compared with that in the control group [2.00 (1.00, 3.00) vs. 2.00 (1.00, 3.00), *p* = 0.08] by the Wilcoxon test. Likewise, the paroxetine group showed a reduced but not significant LOS hospital than the control group [6.00 (5.00, 7.00) vs. 7.00 (6.00, 8.00), *p* = 0.06].

### Subgroup Analysis

We identified 68 patients with hypertension in the matched cohort. The 28-day survival time of HF patients with hypertension in the paroxetine group did not differ from that of the control group (0.88, 95% CI −0.3 to 2.1, *p* = 0.15). In addition, the paroxetine group was associated with a reduced but not significant survival time at a 2,000-day truncation time point (−161 days, 95% CI −917.51 to 595.7, *p* = 0.68).

## Discussion

To the best of our knowledge, this is the first clinical study to investigate the effect of paroxetine therapy on the mortality of HF patients. After matching 38 covariates to reduce underlying confounders, we found that HF patients who received paroxetine in the first 24 h in the ICU did not show statistically significant difference compared to those who did not receive paroxetine in terms of mortality, LOS ICU, and LOS hospital. Although patients treated with paroxetine showed an increased survival time (203 days), this difference was not statistically significant. We also applied several matching methods to strengthen the robustness of the causal inference study, and the results were consistent.

Extensive studies have demonstrated that GRK2 inhibitors, including paroxetine, play a protective role in maintaining cardiac function and improving mortality in animal models by inhibiting GRK2 activity (3, 7, 18). A study showed that small molecule Gbg-GRK2 inhibition initiated 1-week post-injury preserves cardiac contractility and reduces cardiac fibrotic remodeling. It even demonstrated a cardioprotective effect on cardiomyocyte-restricted GRK2 ablated mice, indicating a potential protective role beyond the cardiomyocyte (19). Previous studies have suggested that SSRIs have pleomorphic effects that decrease vasomotor tone and anti-adrenergic effects (20, 21). Furthermore, a recent study suggested that paroxetine may attenuate hypertension-associated cardiac hypertrophy by blocking GRK2-βAR interaction. In hypertensive patients with depression, cardiac remodeling was less severe in those with paroxetine treatment compared with that in those with other types of anti-depressive agents. However, in our subgroup analysis of hypertensive patients, survival time did not differ between the groups. The mean paroxetine administration time of one prescription was relatively short at 5.76 days. This indicates that paroxetine might have no short-term cardiovascular benefits. As proof, the RMST analysis with a relatively long time point (3,000 days) showed a longer survival time in the paroxetine group than in the control group.

People with HF have a significantly increased risk of newly diagnosed depression, and people with depression have a significantly increased risk of developing newly diagnosed HF (22). There were no patients diagnosed with anxiety or depression in either group in our study after matching, so the cofounder regarding SSRIs should be avoided.

Inotropic stimulation has been proven to increase mortality in patients with HF (23). The GRK2 inhibitor could restore catecholamine responsiveness; therefore, it seems to be equivalent to inotropic stimulation. Nevertheless, the coadministration of paroxetine and metoprolol significantly reduced blood pressure and heart rate in spontaneously hypertensive and cardiac hypertrophic rats (18). This phenomenon could be interpreted as GRK2i inhibiting ADRB1 internalization under catecholamine stimulation in cardiomyocytes, which restores cardiac response to ADRB1-blockers, such as metoprolol, ultimately improving the therapeutic effects of β-blockers.

HF-induced cardiac stress promotes catecholamine release to compensate for reduced cardiac output, subsequently increasing GRK activity, and further activating adrenergic signaling to increase output. This cascade leads to irreversible GRK2 upregulation to a point at which therapeutic intervention is required. Ideally, interrupting the worsening circle by introducing GRK2i would help the myocardium to become more responsive to endogenous catecholamine signaling to increase cardiac output. However, it is unknown how much time GRK2i takes to restore myocardium responsiveness to catecholamines. Based on this study, short-term paroxetine administration appears to be insufficient.

### Limitations

As an observational study retrospectively performed on electronic health record data from a single center with limited sample size, a lot of data was missing in the database. We only have the time of death of the patient, but not the events that led to his/her death. Also, there was very little data on BNP, and arguably none on EF, and we listed in the text the percentage of patients who had these variables tested. Our findings should be regarded as preliminary or hypothesis-formulating rather than definitive testing regarding the use of paroxetine in the management of HF patients. Although we matched 38 covariates from different domains of the participants, we still cannot rule out potential confounding variables due to the nature of observational studies. Moreover, multicenter investigations are required to generalize the evidence.

The major limitation of this study was that we did not restrict the maximum duration of paroxetine therapy. Nevertheless, we did not adjust for the year of ICU admission, which is a limitation of the analysis, as practice patterns may have changed during the study period. Some analyses require prospective randomized trials for confirmation.

## Conclusion

In patients with HF, short-term use of paroxetine did not significantly reduce or increase 28-day all-cause mortality. Further large-scale randomized controlled trials are needed to test the use of paroxetine in patients with HF.

## Data Availability Statement

The original contributions presented in the study are included in the article/supplementary material, further inquiries can be directed to the corresponding author/s.

## Ethics Statement

The studies involving human participants were reviewed and approved by the Institutional Review Boards of the Massachusetts Institute of Technology and Beth Israel Deaconess Medical Center. The patients/participants provided their written informed consent to participate in this study.

## Author Contributions

HX, YS, and DL conceived the concept of this study. HX drafted the manuscript. HL, LM, YL, and LW did critical revision of the manuscript for important intellectual content. All authors contributed to the article and approved the submitted version.

## Funding

The Chinese Academy of Medical Sciences funded the present study, CAMS Innovation Fund for Medical Sciences (Grant No. 2018-I2M-1-002), National Key R&D Program of China (Grant No. 2020YFC2003000), and National Natural Science Foundation of China (Grant Nos. 31271097 and 51672030).

## Conflict of Interest

The authors declare that the research was conducted in the absence of any commercial or financial relationships that could be construed as a potential conflict of interest.

## Publisher's Note

All claims expressed in this article are solely those of the authors and do not necessarily represent those of their affiliated organizations, or those of the publisher, the editors and the reviewers. Any product that may be evaluated in this article, or claim that may be made by its manufacturer, is not guaranteed or endorsed by the publisher.
